# Serum Thrombopoietin Levels and Its Relationship With Thrombocytopenia in Patients With Cirrhosis

**DOI:** 10.5812/hepatmon.18556

**Published:** 2014-05-05

**Authors:** Tuncer Temel, Dondu Uskudar Cansu, Halide Edip Temel, Aysegul Harmanc Ozakyol

**Affiliations:** 1 Department of Internal Medicine, Division of Gastroenterology, Faculty of Medicine, Eskisehir Osmangazi University, Eskisehir, Turkey; 2 Department of Internal Medicine, Division of Rheumotology, Faculty of Medicine, Eskişehir Osmangazi University, Eskisehir, Turkey; 3 Biochemistry Department, Pharmacy Faculty, Anadolu University, Eskisehir, Turkey

**Keywords:** Liver Cirrhosis, Thrombocytopenia, Thrombopoietin

## Abstract

**Background:**

Patients with cirrhosis usually have thrombocytopenia in discrete levels. The mechanism of thrombocytopenia is thought as splenic sequestration and destruction of platelets, impaired bone marrow generation and diminished hepatic thrombopoietin synthesis.

**Objectives:**

The aim of this study was to evaluate serum thrombopoietin levels and its relationship with thrombocytopenia at patients with cirrhosis.

**Patients and Methods:**

Ninety–two cirrhotic patients and 45 healthy controls without history or findings of pathologies that can effect thrombopoietin levels were enrolled by simple random sampling to patient and control groups of this case control study performed at Eskisehir-Turkey. Thrombopoietin was measured in serum samples with a solid phase enzyme-linked immune absorbent assay. Additionally, spleen size and volume index were determined.

**Results:**

Platelet counts were lower in patients with cirrhosis (97000 ± 8000/mm^3^) than in healthy subjects (240000 ± 51000/mm^3^, P < 0.001). Significant difference was determined for platelet counts among child A, B and C stages (Child A vs. Child B P < 0.05 Child A vs. Child C P < 0.001–Child B vs. Child C P < 0.05). Serum TPO concentration was higher (69 ± 12 pg/mL) in cirrhotic group than healthy controls (49 ± 9 pg/ml) (P < 0.05). No significant difference in TPO levels were found among the Child A, B and C stages (64 ± 11 pg/mL, 75 ± 13 pg/mL and 68 ± 10 pg/mL, respectively). Spleen size and SVI was significantly higher in the cirrhotic patients than healthy controls (148 ± 14 mm vs. 98 ± 11 mm, P < 0.001-9167 ± 287 cm^2^ vs. 4118 ± 123 cm^2^). Significant difference was determined for spleen size and spleen index among child A, B and C stages (Child A vs. Child B P < 0.05 Child A vs. Child C P < 0.001–Child B vs. Child C P < 0.05). TPO levels were significantly different between cirrhotic patients with platelet levels below 50.000/mm^3^ (n = 16, plt-count: 41000 ± 8300/mm^3^, TPO levels: 73 ± 7 pg/mL) and above 50.000/mm^3^ (n = 76, plt-count: 105000 ± 9500/mm^3^, TPO levels: 65 ± 10 pg/mL) (P < 0.01). In correlation analysis, there was a strong negative correlation between platelet count-spleen size (P < 0.001, r = -0.74) and platelet count–serum TPO levels (P < 0.001, r = -0.71).

**Conclusions:**

Our results suggest that liver cirrhosis does not cause impaired thrombopoietin production even in the late stage of disease. Thrombopoietin has no contribution for the occurrence of thrombocytopenia in cirrhosis; splenic sequestration seems to be the main factor.

## 1. Background

Liver diseases have several effects on distribution, survival and production of hematopoietic elements (1). Thrombocytopenia; one of the fort coming haematological complication of liver cirrhosis; is often defined as a platelet count below 100000 mm3. Platelet levels beneath 50000 mm3 are referred as severe thrombocytopenia. Cirrhotic patients with or without cancers often require numerous medical and/or surgical procedures during diagnosis and therapy. The presence of thrombocytopenia can aggravate surgical or traumatic bleeding and can also significantly complicate routine patient care, such as liver biopsy, antiviral therapy and medically indicated or elective surgery for cirrhotic patients, resulting in delayed or cancelled medical management and affecting the administration of effective treatment for several conditions (2). The mechanism of thrombocytopenia is thought as splenic sequestration and destruction of platelets (3), impaired bone marrow generation and diminished hepatic thrombopoietin (TPO); principal regulator of megakaryo-thrombopoiesis; production (4). The main source of TPO is liver (5). TPO is the major physiological regulator of platelet production. Binding of TPO to its receptor which is expressed on the surface of stem cells, megakaryocyte progenitor cells, megakaryocytes and platelets activates janus kinase/signal transducers and activators of transcription (JAK/STAT) pathways and stimulates thrombopoiesis. TPO levels are correlated with platelet production. (4, 5). Data at the literature about TPO levels and its relation with thrombocytopenia for cirrhotic patients is conflicting. Although in most studies serum TPO levels are decreased in liver cirrhosis, thrombocytopenia is correlated positively with TPO levels and platelet counts are increased after recovery of liver synthesis function; orthotopic liver transplantation; some studies have been reported that serum TPO concentrations are elevated or normal in thrombocytopenic cirrhosis patients, no correlation was determined between TPO levels and thrombocytopenia, and rather than TPO levels, decreased platelet counts are associated with other factors like splenic sequestration (6-20).

## 2. Objectives

The aim of this study was to evaluate serum thrombopoietin levels and its relationship with thrombocytopenia inpatients with cirrhosis.

## 3. Patients and Methods

### 3.1. Patients

Ninety-two cirrhotic patients among all cirrhotic patients who admitted to our clinic between 01.03.2005–01.02.2013 (mean age 58 ± 11 years, 42 females and 50 males) with or without thrombocytopenia and 45 healthy controls (23 females and 22 males, mean age 54 ± 10 years) were enrolled by simple random sampling to patient and control groups of this case control study performed at Eskisehir-Turkey. The number of patient and control groups were determined by two sample t-test power analysis with an expected power of 0.87. Eskisehir Osmangazi University local ethics committee approved the study (28.02.2005 ethics committee acceptance number: 9) and informed consents were obtained. Diagnosis of liver cirrhosis was made according to the clinical and laboratory parameters, while the diagnosis was confirmed by histological examination in 72 of the patients, diagnosis of cirrhosis was established by liver function tests (bilirubin, albumin, international normalized ratio), ultrasound findings (ascites and splenomegaly, dilated portal and splenic veins, presence of portal collaterals and irregular–nodular contours of the liver) at patients with contraindications to liver biopsy. Exclusion criteria were as follows: diagnosis of essential thrombocythemia or history of familial essential thrombocythemia, inherited or acquired bone marrow failure, previous or ongoing application of recombinant hematopoietic growth factors, autoimmune diseases rather than autoimmune hepatitis or history of immune suppressive treatment, diagnosis of a hematopoietic or solid malignancy, antiviral therapy application within last six months. The severity of liver cirrhosis was assessed according to the Child Pugh’s classification (21). The cause of liver cirrhosis was hepatitis B virus (HBV) infection in 22 cases, hepatitis C virus (HCV) infection in 38 cases, alcohol in four cases, autoimmune hepatitis in 10 cases and cryptogenic cirrhosis in 18 cases. Thirty of the patients were at Child A, 32 of the patients were at Child B and 30 of the patients were at Child C stage. Complete blood count, liver function tests including serum albumin levels and prothrombin time were determined at biochemistry and haematology laboratories of Eskisehir Osmangazi University, Medical Faculty. The size of the spleen was measured by ultrasound, and the spleen index was calculated by multiplication of the long and short axes. Characteristics of the patients are summarized at Table 1.

**Table 1.** Characteristics of the Patients ^a,b^

	Child A (n = 30)	Child B (n = 32)	Child C (n = 30)	Cirrhosis Patients (n = 92)
**Age, y**	58 ± 10	58 ± 13	57 ± 9	58 ± 11
**Sex, M/F**	17/13	15/17	14/16	46/46
**Albumin, g/dL**	4.03 0.59	3.04 0.45	2.44 0.36	3.29 0.83
**PT, sec**	15.0 1.45	17.49 2.16	21.12 2.60	17.4 3.10
**Bilirubin, g/dL**	1.29 0.56	1.9 0.87	7.42 4.54	2.98 3.37

^a^ Data are presented as Mean ± SD.

^b^ Abbreviation: PT, Prothrombin time.

### 3.2. TPO Assay

Venous peripheral blood samples were collected and centrifuged at 2500 rpm for 10 minutes. The serum was separated and stored at -75°C. TPO levels were detected by ELISA (Quantikinine, RD Systems, Wiesbaden Nordenstand, Germany) at haematology laboratory.

### 3.3. Statistical Analysis

Statistical analysis was performed using a commercial statistical software package version 13.0 for Windows. Data are shown as the mean ± standard deviation (SD). Means between two groups were compared by using the one-way analysis of variance, (turey post-hoc test) used to evaluate the association between two variables. A value of P < 0.05 was accepted as statistically significant.

## 4. Results

Platelet counts were lower in patients with cirrhosis (97000 ± 8000/mm^3^) than in healthy subjects (240000 ± 51000/mm^3^, P < 0.001). Significant difference was foundfor platelet counts among child A, B and C stages (Child A vs. Child B P < 0.05 Child A vs. Child C P < 0.001–Child B vs. Child C P < 0.05). Serum TPO concentration was higher (69 ± 12 pg/mL) in cirrhotic group than healthy controls (49 ± 9 pg/mL) (P < 0.05). No significant difference in TPO levels were found among the Child A, B and C stages (64 ± 11 pg/mL, 75 ± 13 pg/mL and 68 ± 10 pg/mL, respectively). TPO levels were similar among the patient groups with different etiology. Spleen size and SVI was significantly higher in the cirrhotic patients than healthy controls (148 ± 14 mm vs. 98 ± 11 mm, P < 0.001-9167 ± 287 cm^2^ vs. 4118 ± 123 cm^2^). Significant difference was determined for spleen size and spleen index among child A, B and C stages (Child A vs. Child B P < 0.05 Child A vs. Child C P < 0.001–Child B vs. Child C P < 0.05). TPO levels were significantly different between cirrhotic patients with platelet levels below 50000/mm^3^ (n = 16, plt-count: 41000 ± 8300/mm^3^, TPO levels: 73 ± 7 pg/mL) and above 50.000/mm^3^ (n = 76, plt-count: 105000 ± 9500/mm^3^, TPO levels: 65 ± 10 pg/mL) (P < 0.01). In correlation analysis, there was a strong negative correlation between platelet count-spleen size (P < 0.001, r = -0.74) and platelet count–serum TPO levels (P < 0.001, r = -0.71). Results are summarized at Table 2.

**Table 2.** TPO Levels, PLT Count, Spleen Size and Index in Cirrhosis and Healthy Subjects ^a,b,c,d,e,f,g^

	Child A, (n = 3)	Child B, (n = 32)	Child C, (n = 30)	Liver Cirrhosis, Total, (n = 92)	Healthy Subjects, (n = 45)
**TPO, pg/mL ^c^**	64 ± 11	75 ± 13	68 ± 10	69 ± 12	49 ± 9
**PLT count, mm^3^^d^,^e^**	134358 ± 9208	92416 ± 7607	58136 ± 5476	97000 ± 8000	240000 ± 51000
**Spleen size, cm^2^^f^**	119 ± 14	144 ± 13	175 ± 11	148 ± 14	98 ± 11
**SVI, cm^2^^g^**	6616 ± 216	9363 ± 246	12529 ± 300	9167 ± 287	4118 ± 123

^a^ Data are presented as Mean ± SD.

^b^ Abbreviation: SVI, Spleen volum index.

^c^ Liver Cirrhosis (Total) versus healthy subjects P < 0.05

^d^ Liver Cirrhosis (Total) versus healthy subjects P < 0.001.

^e^ Child A vs. Child B P < 0.05 Child A vs. Child C P < 0.001–Child B vs. Child C P < 0.05.

^f^ Liver Cirrhosis (Total) versus healthy subjects P < 0.001.

^g^ Liver Cirrhosis (Total) versus healthy subjects P < 0.001.

## 5. Discussion

Thrombocytopenia is the most frequent hematologic abnormality in patients with chronic liver disease (22). Prominent causes blamed to take part at thrombocytopenia of cirrhotic patients are decreased plasma TPO levels, accelerated platelet turnover and reduced platelet production.

Thrombocytopenia is generally considered secondary to the increased sequestration and pooling of platelets in enlarged spleen and destruction of platelets (23, 24). However, no exact correlation between the portal pressure and the platelet count has been determined. Moreover thrombocytopenia may persist after splenectomy or portal decompression and return to normal platelet count was observed following liver transplantation (25). As a result diminished thrombocyte production due to TPO; principal regulator of megakaryogenesis and thrombopoiesis, which is predominantly produced by the liver; insufficiency secondary to advanced liver failure is accused as the reason of thrombocytopenia in cirrhosis (26). While some of the studies determined low plasma levels of TPO (6, 11, 13, 15, 18), others evaluated normal or high plasma levels in contrast of decreased thrombocyte levels at cirrhotic patients (5, 9, 20, 26, 27). Bone marrow examinations of cirrhotic patients revealed absolutely normal findings (28, 29).

In our study, cirrhotic patients had higher TPO levels than healthy subjects and a statistically significant negative correlation was determined between platelet counts and serum TPO levels. Circulating TPO levels is regulated through its binding to the TPO receptor; which is mainly expressed on bone marrow megakaryocytes and circulating platelets; rather than the up-regulation or down-regulation of its production (4, 5). While TPO is produced constantly by the liver, kidney, and marrow stroma, its circulating levels depends on the total amount of TPO receptor (25). The observed increase in circulating TPO in our study might be explained by decreased consumption of TPO secondary to thrombocytopenia and contradictory data throughout the literature may be related with ignorance of the TPO receptors at bone marrow.

Proportion of reticulated platelets (RP) in total platelets (%RP) and glycocalicin index (GCI) are indicators of platelet turnover (30). The accelerated platelet turnover in cirrhotic patients indicates an accelerated platelet clearance in the periphery through hypersplenism. Spleen volume index was significantly elevated and platelet counts were significantly reduced in cirrhotic patients than the healthy controls in our study. A strong inverse correlation was observed between platelet count and spleen size in the patients.

The identification of megakaryocytic cells based on morphological studies at the light microscopy is difficult and results are therefore often unreliable especially in cases of elevated platelet turnover. Dual-color immunofluorescence staining and flowcytometry seems to be a much reliable method (25). Therefore, either the analysis of marrow megakaryocytes by conventional light microscopy method or assuming the marrow megakaryocyte density as normal in liver cirrhosis will not be reliable.

In conclusion, cirrhotic thrombocytopenia is a multi-factorial condition but our results suggest that accelerated platelet clearance in the periphery due to splenic sequestration seems to be the main factor for the thrombocytopenia in liver cirrhosis, rather than impaired thrombopoiesis due to TPO insufficiency. Points to clarify seems to be the factors enhancing splenic sequestration and impaired thrombopoiesis.
